# Hyperbaric Oxygen Therapy in Upper Limb Crush Injury: Why and When?

**DOI:** 10.7759/cureus.59146

**Published:** 2024-04-27

**Authors:** Rui Casimiro, Filipa Poleri, Jorge Pinto, Carolina Andresen, Horacio Costa, Horacio Zenha

**Affiliations:** 1 Plastic and Reconstructive Surgery, Centro Hospitalar Vila Nova de Gaia/Espinho, Vila Nova de Gaia, PRT

**Keywords:** hyperbaric chamber, hyperbaric oxygen therapy, crush injury, hand, upper limb

## Abstract

Introduction

In 2016, the European Committee for Hyperbaric Medicine strongly recommended hyperbaric oxygen therapy (HBOT) adjunctive to surgery in post-traumatic crush injuries, initiating as rapidly as possible. For the last 30 years, HBOT has been used in crush injury, but in most cases as a last resort, after skin flaps necrosis or wound bed infection, diminishing its potential benefits as a complementary treatment. It is, therefore, essential to understand how HBOT modulates the outcome of crush injury, and when to use it, since this can be a significant and underused therapeutic weapon that may alter the natural course of these patients.

Methods

Nineteen (n=19) adult patients with upper limb crush injuries underwent adjunctive HBOT, after the initial surgical approach. The measured outcomes included trauma-related acute complications (tissue necrosis and local infection), and late complications (pseudarthrosis and late deep infection).

Results

Only six (n=6) patients started HBOT in the first 24 hours. Four (n=4) patients presented acute complications; in half of those cases (n=2), HBOT was initiated more than 24 hours post-injury. Late complications were observed in three patients, none of which had initiated HBOT in the first 24 hours post-injury.

Conclusions

Either lack of awareness or logistic difficulties, preclude initiating timely HBOT, limiting its potential benefits. It is important to alert all practitioners to the right timing to initiate HBOT in order to improve these patients’ outcomes.

## Introduction

Crush injury includes a broad spectrum of soft tissue and/or bone injury, consequent to kinetic energy transfer from an object to body tissues. In the upper limb, this type of injury is mostly due to motor vehicle crashes, industrial incidents, or person versus vehicle accidents. There is an inverse relation between the severity of injury and the potential for recovery [1], and, as such, complex crush injury patients are submitted to extended hospitalizations and several re-admissions in order to treat multiple complications and sequels. This carries important distress to the patient, long periods of work absence, as well as elevated costs to the health care system. Moreover, upper crush injury requires a higher threshold for amputation compared to the lower limb, and its maintenance is very important for the patient even when there is limited function [2]. There is, therefore, the need to properly manage upper limb crush injury patients in order to minimize the social and economic burden of this condition.

Immediately after trauma, there is activation of a series of cellular and subcellular mechanisms which ultimately lead to tissue damage and eventually to its irreversible destruction, unless there is timely and appropriate treatment. Urgent surgery with cleansing of the wound, debridement of necrotic tissues, repair of damaged vessels, and bone stabilization is usually mandatory, as well as close monitoring of the wound bed as areas of necrosis may take several days or weeks to define [1]. However, mostly in cases of complex injuries, this acute treatment may not suffice to attain the best outcomes.

Hyperbaric oxygen therapy (HBOT) consists of exposing the patient intermittently to 100% oxygen at a pressure greater than the atmospheric pressure at sea level (1 atmosphere absolute, or 1 ATA) [3-5]. During the last five decades, there has been a growing number of indications of HBOT, including crush injury, and multiple clinical trials have documented improvement in soft tissue endurance and bone healing [6-8]. Its employment requires the availability of specialized mono or multi-place chambers.

For the last 30 years, HBOT has been used in crush injury, but in most cases as a last resort, after skin flaps necrosis or wound bed infection [1,9], diminishing its potential benefits as a complementary treatment. It is, therefore, essential to understand how HBOT modulates the outcome of crush injury, and when to use it, since this can be a significant and underused therapeutic weapon that may alter the natural course of these patients.

The pathophysiology of crush injury and its modulation by HBOT will be depicted, as well as a review of the literature about how and when to apply it. A case series of patients with upper limb crush injury and subsequent treatment with HBOT will be presented and discussed.

There are two dominant characteristics of crush injury. The first one is the establishment of a tissue injury gradient [10] immediately after trauma, from the direct impact zone (primary injury) with irreversible tissue destruction to the healthy and spared areas. In between, there is the penumbra area with tissues that may or may not endure depending on timely and appropriate treatment. The second one is the presence of a self-perpetuating cycle of ischemia, hypoxia, and edema [11] that leads to further tissue destruction (secondary injury) in the penumbra area when it is not interrupted. In order not to expand the area of permanent tissue destruction, the therapeutic strategies in crush injury should focus on interrupting this cycle as timely as possible, since over four to seven hours of ischemia will result in irreversible muscle cell injury [12], and even after adequate revascularization there may be further tissue damage by means of ischemia-reperfusion injury [1]. The rationale for employing HBOT in upper limb crush injury is the interruption of this vicious cycle.

Although there is no universally accepted classification of crush injury, the European Committee for Hyperbaric Medicine (ECHM) has adopted the Gustilo and Williams scale to decide when to use HBOT. In its 2016 guidelines, the ECHM strongly recommended HBOT adjunctive to surgery in all post-traumatic crush injuries following open fractures Gustilo type III B and C (type 1 recommendation), based on double-blind controlled randomized studies, although these studies are scarce and with some methodical flaws (level B) [13]. In less severe cases, it is recommended in compromised hosts or when injury-related risk factors are present (type 1 recommendation, Level C).

The need to rapidly block the imbalance between the offer and demand of oxygen in crushed tissues dictates the initiation of treatment as rapidly as possible, ideally in the first 24 hours post-trauma. On the other hand, since its effects are held only for a short time, multiple sessions are required. Although respecting these two principles, the literature is not consensual on the protocols for its employment [7,10,14,15]. The protocols may differ in the time and number of sessions but all of them ultimately agree that HBOT should not delay any necessary surgical intervention and the decisions regarding surgical management should be made independently, whether HBOT is provided.

## Materials and methods

Study design

A retrospective case series was conducted to assess the outcomes of patients with upper limb crush injuries treated with hyperbaric oxygen therapy (HBOT) between April 2014 and May 2015. The study was conducted at Vila Nova de Gaia Hospital Center (CHVNG) and Matosinhos Local Health Unit (USLM) in Portugal. The study was approved by the institutional review boards of both hospitals.

Patient selection/inclusion and exclusion criteria

Patients were included if they met the following criteria: adults with upper limb crush injury, classified as Gustilo III, admitted to the emergency room of CHVNG or USLM between April 2014 and May 2015 and observed by the Plastic and Reconstructive Surgery specialty.

Patients were excluded if they had undergone amputations due to life-threatening systemic complications. 

For criteria establishment, we considered a total of five studies, all indexed in relevant journals. The sample calculation was 19 (n=19) patients according to these criteria.

Treatment protocol

After receiving standard trauma care in the admitting hospital, eligible patients underwent HBOT at USLM. Those admitted to CHVNG were transported to USLM by ambulance for scheduled treatments. The standard HBOT protocol for crush injuries involved a minimum of two sessions in the first three days, followed by daily sessions until a total of 12 sessions were completed. The total number of sessions could be adjusted based on clinical judgment or logistical constraints. HBOT was administered in a multi-place chamber with each session lasting 90 minutes, during which patients breathed 100% oxygen at 2.1 or 2.4 atmospheres absolute (ATA).

Outcome measures

The primary outcomes measured included trauma-related acute complications, such as significant tissue necrosis requiring additional surgical debridement and local infections necessitating systemic antibiotics. Late complications, including pseudarthrosis and late deep infections, were also assessed. Additionally, recovery and quality of life were evaluated using the upper limb component of the Short Musculoskeletal Function Test, conducted at least six months post-injury.

Data collection

Data regarding acute complications were obtained from clinical records, while phone interviews were conducted to assess late complications and functional recovery.

Statistical analysis

Descriptive statistics were used to summarize patient demographics, injury characteristics, treatment details, and outcomes. Continuous variables were presented as means with standard deviations or medians with interquartile ranges, depending on the distribution. Categorical variables were summarized as frequencies and percentages. Subgroup analyses were performed when appropriate. 

Ethical considerations

This study was conducted in accordance with the principles of the Declaration of Helsinki. Patient confidentiality was maintained throughout the study, and informed consent was obtained from participants or their legal guardians where necessary.

Limitations

Limitations of the study included its retrospective design, potential selection bias, and reliance on medical records and phone interviews for data collection. Additionally, the generalizability of the findings may be limited to similar healthcare settings and patient populations.

## Results

A total of 19 patients were considered eligible, 10 from CHVNG and nine from USLM (Table 1). The patients were mostly male (n=17 vs n=2) with a median age of 32 years (range: 18 to 68 years). Phone interviews were conducted on nine of those patients.

**Table 1:** Detailed patients’ data. M: male; F: female. HBOT: Hyperbaric oxygen therapy.

Age (years)	Sex	Cause of injury	Anatomic location	Gustilo classification	Timing of initial surgery	Primary amputation	1^st^ HBOT session (days)	Number of HBOT sessions	HBOT related complications	Additional surgical debridement	Secondary amputation	Infection	Late complications	Functional outcome (%)
62	F	Industrial accident	Hand	IIIC	< 24h	No	7	3	-	1	Yes	Yes	No	77.70/100
65	M	Motor vehicle crash	Hand and forearm	IIIB	< 24h	No	16	26	-	0	No	No	Yes	87.00/100
20	M	Industrial accident	Hand	IIIC	< 24h	No	0	24	Symptomatic hypoglycemia	4	Yes	Yes	-	-
59	M	Industrial accident	Hand	IIIA	< 24h	No	6	8	-	0	No	No	No	17.00/100
22	M	Motor vehicle crash	Forearm	IIIC	< 24h	No	2	6	-	0	No	No	No	3.00/100
68	M	Industrial accident	Hand	IIIA	< 24h	No	2	5	-	1	Yes	Yes	No	82.00/100
56	M	Industrial accident	Hand	IIIC	< 24h	Yes	2	8	-	0	No	No	Yes	74.00/100
24	M	Industrial accident	Hand	IIIA	< 24h	Yes	1	19	-	0	No	No	-	-
21	M	Industrial accident	All upper limb segments	IIIB	< 24h	No	24	29	-	0	No	No	Yes	24.00/100
32	F	Domestic accident	Hand	IIIA	< 24h	No	3	19	-	0	No	No	-	-
60	M	Industrial accident	Hand	IIIA	< 24h	No	12	52	-	0	No	No	-	-
27	M	Industrial accident	Hand	IIIC	< 24h	No	2	6	-	1	Yes	Yes	-	-
31	M	Industrial accident	Hand	IIIA	< 24h	No	6	3	-	0	No	No	-	-
64	M	Industrial accident	Hand	IIIA	3 days	Yes	0	5	-	1	Yes	No	-	-
55	M	Industrial accident	Hand	IIIB	< 24h	Yes	2	8	Tympanic membrane rupture	0	No	No	No	57.10/100
54	M	Industrial accident	Elbow	IIIB	< 24h	No	1	8	-	0	No	Yes	No	33.90/100
27	M	Industrial accident	Hand	IIIC	< 24h	No	3	4	-	0	No	No	-	-
31	M	Industrial accident	Hand	IIIB	< 24h	No	0	5	-	0	No	No	-	-
18	M	Industrial accident	Hand	IIIB	< 24h	Yes	0	3	-	0	No	No	-	-

Injury characteristics

Industrial accidents were the main cause of injury (n=16), with only two patients admitted for car vehicle crashes and one domestic accident. At admission, there were seven Gustilo IIIA, six Gustilo IIIB, and six Gustilo IIIC injuries. The hand was the most affected segment, with 17 patients presenting hand injury, two of them with forearm and upper arm lesions as well. One patient had trauma to the elbow alone and another had injuries limited to the forearm. Seven patients presented injury in the dominant upper limb.

Emergency surgery

All patients were submitted to surgery with wound cleansing and debridement, bone fracture reduction and fixation, fasciotomy, and vascular, nervous, and tendon repair as needed. There was a need for digital amputation in the emergency setting in five patients. All but one patient had surgery in the first 24 hours post-trauma.

Hyperbaric oxygen therapy sessions

All patients were submitted to HBOT sessions, starting a median of two days after trauma (range: 0 to 24 days). Only six patients started treatment in the first 24 hours. There was a 16-day delay for a polytrauma patient who had to remain in an intensive care unit for two weeks. One patient had the sessions initiated only 24 days post-injury since there was a logistic and administrative deferral. The patients completed a median of eight HBOT sessions (range: 3 to 52 sessions).

There were records of HBOT-related complications in two patients, one symptomatic hypoglycemia and one barotrauma with tympanic membrane perforation, that resolved without the need for invasive treatment.

Outcomes

Overall, six patients presented acute complications (Table 2). In half of those cases, HBOT was initiated more than 24 hours after injury, and they were submitted to a median of 5.5 sessions (range: 3 to 24 sessions). In specific, infection-demanding intravenous antibiotics were developed in five patients. Tissue necrosis with the need for further surgical debridement was reported in five patients, all of them with subsequent digital amputation. Only one patient required more than one supplementary surgery. In one patient, initial surgical debridement had been delayed for three days. Regarding injury classification, Gustilo IIIC presented a 50% (n=3) acute complication rate, with 16.70% (n=1) in IIIB patients and 28.60% (n=2) in IIIA fracture patients.

**Table 2:** Acute and subacute/late complications.

Acute complications				
	All patients (n=19)	Gustilo IIIA (n=7)	Gustilo IIIB (n=6)	Gustilo IIIC (n=6)
Overall complications	31.60% (n=6)	28.60% (n=2)	16.70% (n=1)	50.00% (n=3)
Tissue necrosis only	5.25% (n=1)	14.30% (n=1)	-	-
Local Infection only	5.25% (n=1)	-	16.70% (n=1)	-
Both	21.10% (n=4)	14.30% (n=1)	-	50.00% (n=3)
Subacute/late complications				
	All patients (n=9)	Gustilo IIIA (n=2)	Gustilo IIIB (n=4)	Gustilo IIIC (n=3)
Overall complications	33.30% (n=3)	-	50.00% (n=2)	33.00% (n=1)
Late deep infection only	-	-	-	-
Pseudarthrosis only	22.20% (n=2)	-	25.00% (n=1)	33.00% (n=1)
Both	11.10% (n=1)	-	25.00% (n=1)	-

As for sub-acute or late complications, three out of nine patients presented pseudarthrosis and/or late deep infection (Table 2). One patient presented both late complications, while two others presented with pseudarthrosis only. None of those patients had initiated HBOT in the first 24 hours after injury. Regarding injury classification, all these patients presented Gustilo IIIB or IIIC fractures.

Concerning functional outcome, evaluated with the upper limb component of the Short Musculoskeletal Function Test at least six months after injury, the interviewed patients presented a median score of 57.10/100 (range: 3 to 87), but only two were able to return to their previous work activity.

## Discussion

The evidence on the usefulness of HBOT may be stronger for chronic than acute wounds and there are few studies regarding upper limb crush injury patients, most of them case reports, but there is consensus that HBOT appears beneficial in those patients [7,8,16]. Its effectiveness lies in the ability to overcome the ischemia and hypoxia that characterize crush injury [3,4].

The guidelines presented in 2016 by the ECHM state very clearly that all Gustilo fractures type III B and C should be submitted to complementary HBOT [13] as rapidly as possible (Table 3). The sessions should begin no more than a day after trauma. However, it is not always feasible to initiate this treatment in the first hours post-injury because of the limited availability of specialized facilities and transportation logistics [3], as well as the lack of awareness by many professionals. The growing number of chambers worldwide may overcome this difficulty. However, even with a hyperbaric chamber at a 15-kilometer distance, the initial treatment of the presented cases was optimal only in less than one-third of cases. In patients in which HBOT was initiated several days after injury, it is discussable if these patients’ outcome is related at all to its employment, but the sessions were maintained anyway since there was a favorable clinical response. In other patients, the delay was probably due to a lack of awareness of the need for early and timely initiation of the protocol. In retrospective analysis, half the patients with acute complications initiated treatment more than 24 hours after injury, when they had already developed digital venous congestion, so the maximum benefit of a hyperbaric environment was certainly not attained. One must regard as well that none of the patients who presented late complications had initiated timely HBOT sessions. 

**Table 3:** European Committee for Hyperbaric Medicine guidelines for HBOT in crush injury. Type 1 recommendation: Strongly recommended. The Jury considers the implementation of the recommendation of critical importance for final outcome of the patient/quality of practice/future specific knowledge. HBOT: Hyperbaric oxygen therapy.

Gustilo type	Injury	HBOT
I	Small laceration from inside to outside (< 1 cm)	No HBOT
II	Laceration more than 1 cm long without or with minimal soft tissue injury	No HBOT
IIIA	Crush injury component; adequate soft tissue coverage	Can be used in compromised hosts (Type 1 recommendation)
IIIB	Crush injury component; inadequate soft tissue to cover bone and close wound	HBOT (Type 1 recommendation)
IIIC	Crush injury component with arterial injury	HBOT (Type 1 recommendation)

The patient transport to HBOT sessions may also be particularly challenging, especially concerning an unstable patient. In multi-place chambers, oxygen administration is applied through masks, hoods, or endotracheal tubes, so it is possible for the patient to be accompanied by specialized personnel [4]. One has, however, to carefully exclude absolute and relative contraindications to its employment and weight risks versus benefits of HBOT and transport [17].

As for the number of sessions, only one out of six patients with acute complications completed the twelve sessions. Although this could indicate that the remaining patients did not go through a complete treatment, this is more probably due to the clinical judgment determination that no more sessions were needed after having amputated and debrided the tissues at risk. More trials are needed to determine if the twelve sessions are indeed necessary since each session carries a high financial burden. Nonetheless, according to the ECHM, the cost of the use of adjunctive HBOT will be at least compensated by the decrease in morbidity in these patients [13]. In the long term, even if an amputation surgery has a lower direct cost, the rehabilitation period and work absence will result in a heavier financial burden.

Concerning side effects, several studies state that, in specialized centers, complications are rare and usually well tolerated [9] and it's safe when used in standard protocols [4]. This series presented one hypoglycemia and one case of middle ear barotrauma, which constitute the most common complications described in the literature, making HBOT a rather safe treatment [3,4,10].

Regarding clinical outcomes, our overall acute complication rate and the need for additional amputation were higher compared to other studies using HBOT, namely the work of Matos et al. [18] where HBOT was applied to Gustilo III fractures with a successful outcome in 91.30%, with only 13% requiring amputations, and Bouachour et al. [7] which mentions complete healing without the need for further surgery in 94%. This may be due to the aforementioned difficulties precluding a timely treatment. Nevertheless, the one-third acute complication rate verified for Gustilo IIIB and IIIC fractures in this case series, was still lower than the 44 to 50% described in the literature for the same fracture type without HBOT [19,20].

Unsurprisingly, there seems to be a correlation between injury severity and complications. Half the patients with acute complications presented Gustilo IIIC fractures and all of those developed both significant tissue necrosis and infection. Not only the lesion severity but also the presence of necrotic tissue with impaired tissue irrigation and infection resistance, as well as the higher number of surgeries, may explain this association between infection and additional debridement.

This study presents several limitations including being a retrospective case series, involving only two different hospital centers. The small sample size precluded statistical analysis regarding other factors potentially affecting the outcome, namely age, smoking habits, and comorbidities. Not having measured transcutaneous oxygen pressure to guide the indication and evolution of treatment constitutes another limitation to this study.

## Conclusions

Physiological and biochemical pathways, as well as the current guidelines from the ECHM, strongly favor the use of HBOT in upper limb crush injury. Nevertheless, there is still a great gap between these theoretical principles and clinical applications. The authors believe this small case series mirrors the current reality of HBOT practice. Although HBOT is currently recommended in crush injuries, either the lack of awareness or logistic difficulties, frequently preclude initiating treatment in the first 24 hours after injury and limit its potential benefits. The ongoing multicentric prospective randomized trial concerning this matter is due publication in 2018 and may further clarify HBOT's usefulness and help to alert all practitioners regarding the right timing to initiate HBOT and the need for developing institutional HBOT protocols, in order to improve these patients’ outcomes.
